# A Rare Presentation of Rhodococcus Equi Bacteremia as a Result of Right Upper Arm Cellulitis: A Case Report and Literature Review

**DOI:** 10.7759/cureus.38295

**Published:** 2023-04-29

**Authors:** Keana-Kelley D Swanner, Riya Patel, Thuy T Nguyen, Felicia N Patel, Raul Magadia, Ahmad O Rifai, Margaret Davenport

**Affiliations:** 1 College of Medicine, Alabama College of Osteopathic Medicine, Dothan, USA; 2 Internal Medicine, Alabama College of Osteopathic Medicine, Dothan, USA; 3 Department of Infectious Diseases, Regional Medical Center, Anniston, USA; 4 Nephrology, The Virtual Nephrologist, Panama City Beach, USA; 5 Internal Medicine, Regional Medical Center, Anniston, USA

**Keywords:** peritoneal dialysis (pd), immunocompromised state, end stage renal disease (esrd), bacteremia, rhodococcus

## Abstract

*Rhodococcus equi* is an emerging opportunistic pathogen in immunocompromised patients. Owing to its resemblance to *Mycobacterium*, *Nocardia*, and *Corynebacterium*, *R. equi *is frequently misdiagnosed as a contaminant, which can result in treatment delays.

A 65-year-old man with a history of end-stage renal disease (ESRD) presented to the emergency room with pain and increased swelling in his right upper extremity. Shortly after he arrived in the emergency room, his condition deteriorated. Intravenous vancomycin was administered after collecting blood cultures. The blood cultures grew *Rhodococcus equi, and *oral azithromycin and oral rifampin were added for a 14-day course of treatment. The patient recovered without any further complications and was subsequently discharged home.

*R. equi* is a partially acid-fast actinomycete that spreads through contact with grazing animals and contaminated soil. *R. equi *invades macrophages to survive and causes infection within a host. In this particular case, the patient worked on a farm taking care of goats. He was exposed to the bacteria after falling and sustaining multiple lacerations to the right arm.

This case is unique due to the development of bacteremia with *R. equi*, an uncommon cause of bacteremia that led to cardiopulmonary arrest. The treatment with oral azithromycin combined with oral rifampin and intravenous vancomycin was effective for the complete resolution of the infection.

## Introduction

*Rhodococcus equi* is an emerging opportunistic pathogen that affects immunocompromised patients, including those with HIV/AIDS, organ transplant recipients, and cancer patients [1]. Originally an animal pathogen found in foals with pneumonia, *R. equi *has increasingly infected humans since the first reported case in an autoimmune hepatitis patient receiving immunosuppressive medications in 1967 [2-4]. *R. equi *causes a spectrum of diseases, primarily pulmonary syndromes. Extrapulmonary infections that occur without concurrent pulmonary diseases account for about 25% of all reported cases. These include cellulitis resulting from wound inoculation, peritoneal catheter-related infections, lymphadenitis, and isolated bacteremia [5].

Formerly known as* Corynebacterium equi,* *R. equi *belongs to the *Nocardiaceae* family, which also includes *Mycobacterium*, *Nocardia*, *Corynebacterium*, and *Gordonia* [2]. It is a gram-positive, partially acid-fast, *asporogenous coccobacillus* that can change shape depending on growth conditions [1,6].

This non-motile, obligate aerobe grows well in the soil at temperatures ranging from 10° to 40°^ ^C, as natural nutrients are readily available from the manure of grass-grazing animals [1,3, 7-8]. It has an optimal growth environment in the intestines of grazing animals [1,8]. *R. equi *causes infection by invading macrophages and surviving inside of them [4].

We report the case of a 65-year-old man who was immunocompromised due to end-stage renal disease. He presented with *R. equi *cellulitis of the right upper extremity, which led to bacteremia, progressed to sepsis, and resulted in cardiopulmonary arrest. The patient was treated with intravenous vancomycin and oral azithromycin combined with oral rifampicin and recovered without further complications.

## Case presentation

**Figure 1:**
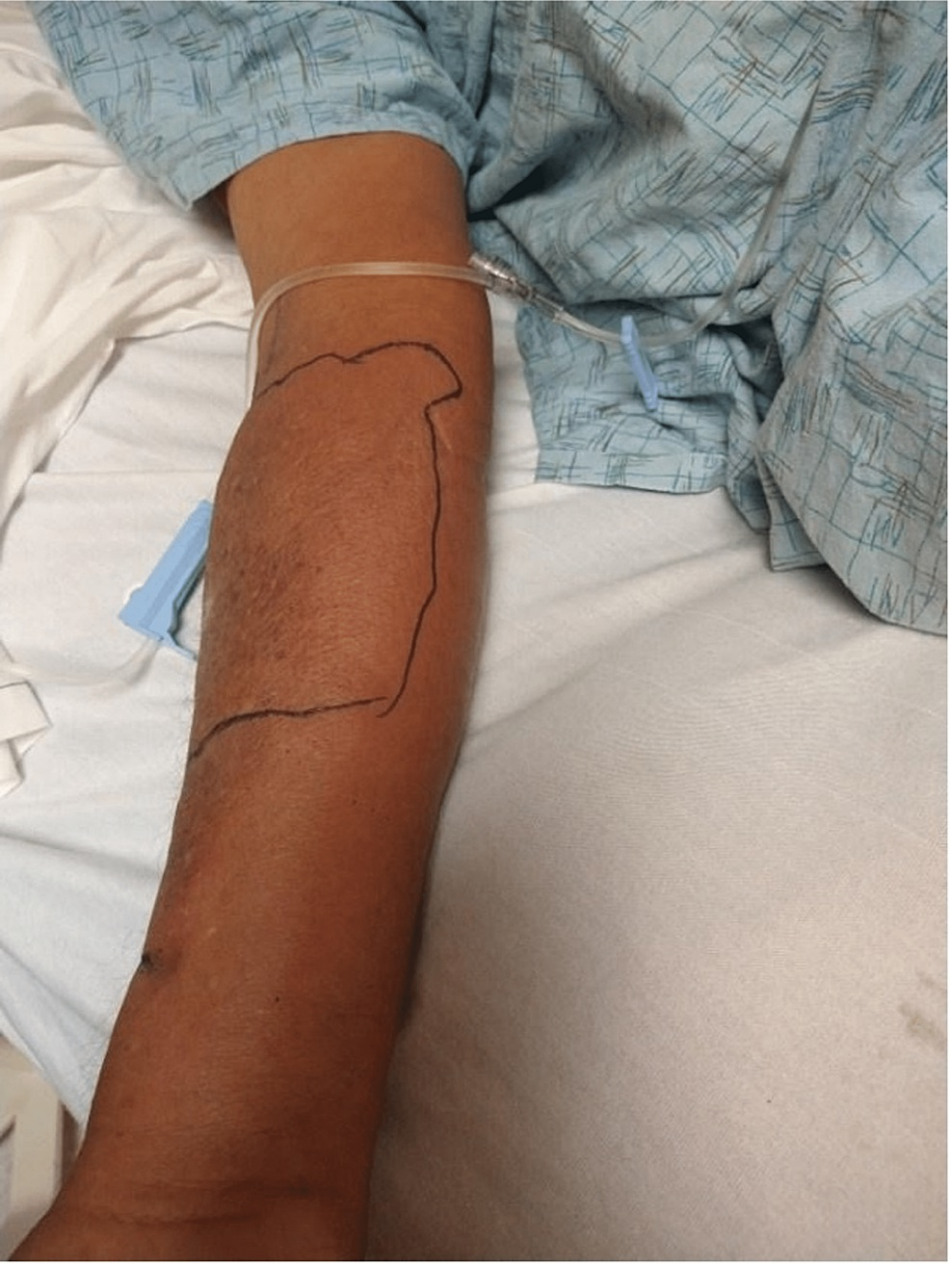
Right upper extremity cellulitis upon ER presentation.

A 65-year-old man presented to the emergency room (ER) with swelling on his right forearm after swatting a spider off his arm, which rapidly progressed (Figure 1). While in the ER, the patient developed dyspnea and became tachycardic. The patient has a past medical history of hypertension, coronary artery disease with recent three-vessel coronary artery bypass grafting, myocardial infarction, congestive heart failure, type 2 diabetes mellitus, and end-stage renal disease (ESRD) on peritoneal dialysis for the past three years. He resides with his wife in their country home, where they keep chickens, goats, cats, and one dog.

His initial vital signs demonstrated a temperature of 97.9 F, a heart rate of 104 beats per minute, a blood pressure of 137/93 mmHg, a respiratory rate of 18 breaths per minute, an oxygen saturation of 96% in room air, and a body mass index (BMI) of 29.3. The cardiac exam revealed tachycardia with a regular rhythm and no murmurs, and lung auscultation was clear bilaterally. The patient had a peritoneal catheter in the left lower quadrant, but there was no skin discoloration or swelling in the surrounding area. The examination of the patient’s upper extremity revealed diffuse erythema and intense swelling on the volar surface of the right forearm extending from the wrist to the elbow (Figure 1). The area was extremely tender to light palpation, but there was no fluctuance or subcutaneous crepitus.

The pertinent patient’s initial blood work is shown below (Table 1). An EKG showed evidence of a right bundle branch block, and there were no acute findings on a chest radiograph. The patient was admitted to the inpatient hospital unit and started on empiric intravenous vancomycin after appropriate blood cultures were collected.

**Table 1:** Patient's laboratory values upon emergency room presentation.

Laboratory Data		Reference Range
WBC (White Blood Count)	11.4 x 10^9^/L	4.5-11 x 10^9^/L
Hgb (Hemoglobin)	12.9 g/dL	13.5-17.5 g/dL
Hct (Hematocrit)	40.20%	41%-50%
BUN (Blood Urea Nitrogen)	53 mg/dL	5-20 mg/dL
Creatinine	8.24 mg/dL	0.7-1.3 mg/dL
High-sensitivity Cardiac Troponin	183.2 ng/L	<14 ng/L

The following day, the patient became hypotensive, and he was transferred to the intensive care unit (ICU), where he received an intravenous vasopressor of norepinephrine. Both cultures from both sets grew gram-positive *cocci*.

**Figure 2:**
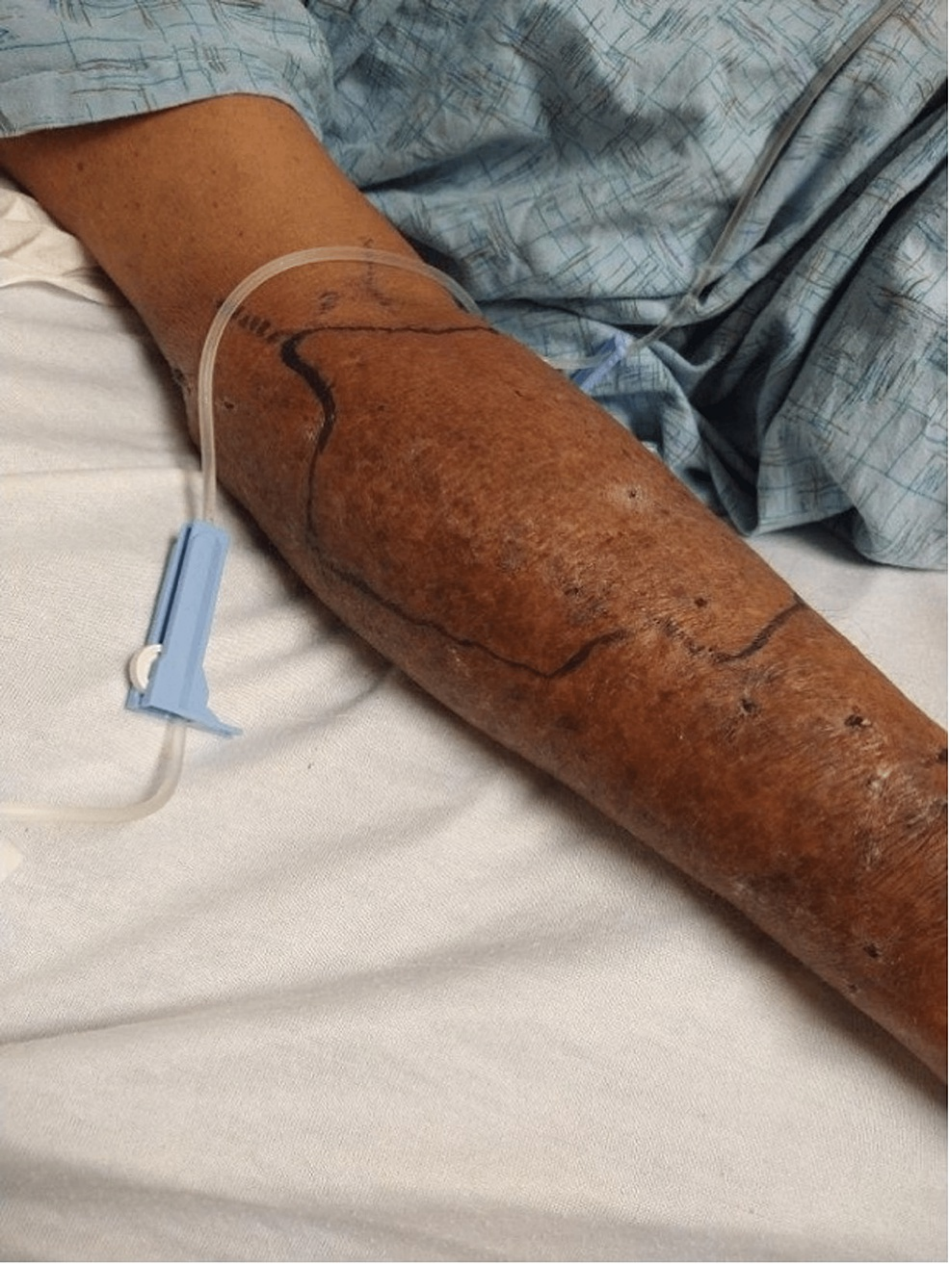
Cellulitis with worsening skin changes on the fourth day of admission, before successful antibiotic treatment.

On the fourth hospital day, two sets of blood cultures confirmed *Rhodococcus equi *bacteremia. Oral azithromycin and rifampicin were added for 14 days to the existing regimen of intravenous vancomycin [5]. His cellulitis gradually improved after starting the triple antibiotic regimen (Figure 2).

On the night before discharge from the ICU, the patient suffered a cardiopulmonary arrest and required intubation and mechanical ventilation. His lactate level was elevated at 4.7 mmol/L (0.5-2.2 mmol/L). Despite this event, his antibiotic regimen of intravenous Vancomycin, oral Azithromycin, and oral Rifampicin was continued without modification. The workup ruled out pulmonary embolism and cardiac ischemia as causes. After four days of support, the patient eventually stabilized and was successfully extubated.

After completing 14 days of intravenous vancomycin, oral azithromycin, and oral rifampin, his repeat blood cultures came back negative.

## Discussion

In our case, the patient was a farmer who regularly cared for his animals, including goats. He was exposed to soil and manure daily. While tending to the goats, he sustained a laceration on his right forearm, which may have resulted in the inoculation with *R. equi*. Due to its rarity, *R. equi* was not initially considered in the ER. Initially, a spider bite was considered the probable cause due to the patient's reported history of spider exposure. Therefore, only intravenous vancomycin was administered, as the risk of methicillin-resistant *staphylococcus* (MRSA) was high. However, after the blood cultures confirmed *R. equi,* oral azithromycin, and oral rifampicin were added to the intravenous vancomycin regimen and continued for 14 days.

Our patient was considered immunocompromised due to his end-stage renal disease diagnosis. This condition causes markedly elevated uremic toxins that impose oxidative stress on the body, leading to the release of more proinflammatory cytokines [9].

This environment of end-stage renal disease disturbs both innate and adaptive immunity by decreasing the number and function of macrophages, dendritic cells, neutrophils, and natural killer cells [10]. Furthermore, the patient had heart disease and severe infection, which are the two main causes of mortality in ESRD patients [9].

Vancomycin was initially chosen as a treatment for the patient based on his report of a spider bite. MRSA infections are common in spider bites, specifically spiders that are endemic to the southeast where the patient resides, making vancomycin a suitable choice of treatment [11].

The patient described the spider as a “small black spider,” which could be assumed to be a black widow spider. Black widow spiders can cause hypocalcemia, leading to muscle cramps and stiffness [12]. However, our patient did not exhibit hypocalcemia or related symptoms, such as tetany or cramps. This ruled out the possibility of a black widow spider bite. 

Intravenous piperacillin and tazobactam were not used, even though the patient was diabetic and had an increased risk of infection with anaerobic and gram-negative bacteria [13]. This was because the initial assumption was that the patient had most likely suffered from an MRSA infection. There was no evidence of subcutaneous air on physical examination at the time of presentation; plain films were not taken [13].

Gas-producing organisms are typically anaerobes, but since the physical examination did not show any signs of subcutaneous crepitus, they were ruled out as potential pathogens. Therefore, intravenous piperacillin and tazobactam were not initiated.

The identification of the pathogen in our patient was challenging due to several factors. First, he was exposed to farm animals daily, particularly his goats. Second, he had lacerations that were in contact with barn soil and manure. Third, he reported swatting a spider before presenting to the ER. Finally, his extensive comorbidities had compromised his immunity, making him more susceptible to infections.

Previous cases of *R. equi *infections, both in immunocompromised and immunocompetent patients, have presented a challenge in selecting antibiotics that are effective against the organism while also preventing antibiotic resistance [9].

Due to *R. equi’s *ability to survive in macrophages and cause bacteremia in the host, it has been historically preferred to use at least two antibiotics to provide effective treatment [9]. Table 1 provides examples of past cases of *R. equi *in immunocompetent hosts, including their respective treatments and outcomes. (Table 2) Quinolines, macrolides, and rifampicin are the agents of choice against the intracellular form of *R. equi*. However, the overuse of rifampin and macrolides in horse breeding farms in the United States over the last few decades may have contributed to the emergence of dual therapy-resistant strains [7,14-22].

**Table 2:** A few of the reported cases of skin and soft tissue infections from R. equi

Exposure	Localization	Immune Status	Treatment	Outcome	References
Not found	Right breast cellulitis	Immunocompetent	Moxifloxacin and Rifampin	Recovered	Sandkovsky et al., 2011 [14]
Farm animals, soil, and manure	Left breast granulomatous mastitis	Immunocompetent	Ciprofloxacin and Azithromycin	Recovered	Nath et al.,2013 [15]
Farm	Temporal scalp wound inflammation	Immunocompetent	Erythromycin and Rifampicin; Surgical debridement	Recovered	Nasser & Bizri, 2001 [16]
Road and Soil	Knee	Immunocompetent	Surgical debridement and irrigation	Recovered	Muller et al., 1988 [17]
Not found	Left foot ulcer	Immunocompetent	Surgical excision; azithromycin and rifampicin	Recovered	Stewart et al., 2019 [4]

For better patient outcomes and a decrease in relapses, it is essential to promptly diagnose an *R. equi* infection and initiate dual antimicrobial therapy. The duration of treatment and choice of regimen should be based on the patient’s immune status. For immunocompromised patients, it is recommended to increase the duration of antimicrobial therapy.

## Conclusions

*Rhodoccoccus equi *is an emerging opportunistic pathogen that affects immunocompromised patients. Given *R. equi’s* ability to survive in macrophages, it is crucial to use at least two antibiotics for treatment. Overuse of antibiotics to treat *R. equi *in horse breeding farms has led to the emergence of dual therapy-resistant strains. The current agents of choice for treatment include quinolines, macrolides, and rifampicin. This case highlights the development of occult bacteremia and cardiopulmonary arrest in an immune-compromised patient with *R. equi-*associated cellulitis and bacteremia.
